# Lymph Node Metastases and Prognosis in Left Upper Division Non-Small Cell Lung Cancers: The Impact of Interlobar Lymph Node Metastasis

**DOI:** 10.1371/journal.pone.0134674

**Published:** 2015-08-06

**Authors:** Hiroaki Kuroda, Yukinori Sakao, Mingyon Mun, Hirofumi Uehara, Masayuki Nakao, Yousuke Matsuura, Tetsuya Mizuno, Noriaki Sakakura, Noriko Motoi, Yuichi Ishikawa, Yasushi Yatabe, Ken Nakagawa, Sakae Okumura

**Affiliations:** 1 Department of Thoracic Surgical Oncology, Japanese Foundation for Cancer Research, Cancer Institute Hospital, Tokyo, Japan; 2 Department of Thoracic Surgery, Aichi Cancer Center Hospital, Nagoya, Japan; 3 Department of Pathology, Japanese Foundation for Cancer Research, Cancer Institute Hospital, Tokyo, Japan; 4 Department of Pathology and Molecular Diagnosis, Aichi Cancer Center Hospital, Nagoya, Japan; Peking University People Hospital, CHINA

## Abstract

**Background:**

Left upper division segmentectomy is one of the major pulmonary procedures; however, it is sometimes difficult to completely dissect interlobar lymph nodes. We attempted to clarify the prognostic importance of hilar and mediastinal nodes, especially of interlobar lymph nodes, in patients with primary non-small cell lung cancer (NSCLC) located in the left upper division.

**Methods:**

We retrospectively studied patients with primary left upper lobe NSCLC undergoing surgical pulmonary resection (at least lobectomy) with radical lymphadenectomy. The representative evaluation of therapeutic value from the lymph node dissection was determined using Sasako’s method. This analysis was calculated by multiplying the frequency of metastasis to the station and the 5-year survival rate of the patients with metastasis to the station.

**Results:**

We enrolled 417 patients (237 men, 180 women). Tumors were located in the lingular lobe and at the upper division of left upper lobe in 69 and 348 patients, respectively. The pathological nodal statuses were pN0 in 263 patients, pN1 in 70 patients, and pN2 in 84 patients. Lymph nodes #11 and #7 were significantly correlated with differences in node involvement in patients with left upper lobe NSCLC. Among those with left upper division NSCLC, the 5-year overall survival in pN1 was 31.5% for #10, 39.3% for #11, and 50.4% for #12U. The involvement of node #11 was 1.89-fold higher in the anterior segment than that in the apicoposterior segment. The therapeutic index of estimated benefit from lymph node dissection for #11 was 3.38, #4L was 1.93, and the aortopulmonary window was 4.86 in primary left upper division NSCLC.

**Conclusions:**

Interlobar node involvement is not rare in left upper division NSCLC, occurring in >20% cases. Furthermore, dissection of interlobar nodes was found to be beneficial in patients with left upper division NSCLC.

## Introduction

Lobectomy with systemic lymphadenectomy is a standard treatment for resectable non-small cell lung cancer (NSCLC). Management of lymph node negative (cN0) patients poses a clinical dilemma; approximately 10% of cN0 patients in our institution have hilar or mediastinal lymph node involvement. Although there is evidence that advanced NSCLC patients benefit from adjuvant chemotherapy after complete pulmonary resection [1–3], the value of radical lymph node dissection remains undetermined. Likewise, the long-term outcomes associated with significant radical mediastinal lymph node dissection with lobectomy remain controversial. Two major retrospective, randomized studies have reported contradictory results [4, 5]. Wu et al. reported that mediastinal lymph node dissection was essential for both accurate staging and improved survival compared with that for sampling alone [4], whereas the American College of Surgery Oncology Group Z0030 trial reported that mediastinal lymph node dissection does not improve survival in patients with early stage NSCLC [5]. Some retrospective studies have published nodal spread patterns according to tumor location [6, 7]; consequently, modified lymph node dissection with selective lymphadenectomy is becoming increasingly prevalent.

Several recent retrospective studies reported that the prognosis of segmentectomy is equivalent to that of lobectomy in patients with cT1N0M0 NSCLC despite short survival intervals [8–10]. Nomori et al. reported on radical segmentectomy for cT1N0M0/pN0 NSCLC [11]; they extensively dissected the hilar mediastinal lymph nodes and used radioisotopes to identify the sentinel node. When metastasis was diagnosed at the primary sentinel node (numbers 10–13) site according to the definition of the Committee of the International Union against Cancer [12, 13] and in lobe-specific nodes, complete lobectomy was performed instead of segmentectomy. In this manner, the precision of segmentectomy was increased by using radioisotopes to detect the sentinel node [14].

The aim of this study was to review the prevalence of lymph node involvement according to each mediastinal hilar region in patients with left upper lobe NSCLC. In addition, we investigated which hilar lymph node is involved in left upper division NSCLC. Finally, because complete dissection interlobar lymph nodes can be difficult due to variations in the divergence style of the lingular artery and vein during left upper segmentectomy, we also investigated whether abbreviation of interlobar lymph node dissection was possible in patients who underwent left upper division segmentectomy.

## Patients and Methods

We retrospectively studied 417 patients (237 men, 180 women) with primary left upper lobe NSCLC. All patients were required to have undergone left upper resection (at least lobectomy) with lymphadenectomy (more than ND2–1) between January 1995 and December 2010. Participants were enrolled at either the Aichi Cancer Center Hospital or the Japanese Foundation for Cancer Research, Cancer Institute Hospital. We excluded patients who had received preoperative chemotherapy and radiotherapy and those who underwent lymph node sampling.

The clinical data for staging were obtained by computed tomography (CT) scans of the chest and abdomen, magnetic resonance imaging of the head, abdominal ultrasound, bone scintigraphy, and/or positron emission tomography. Tumors were staged according to the TNM classification system (seventh edition) [13]. Pathological examination was based on the 2004 World Health Organization classification [14]. The lymph node location was defined according to the Committee of the International Union against Cancer [12] guideline; # indicates lymph node number and (+) and (−) represent the positive and negative status of the node, respectively, where indicated. To define the dominant segment, we identified the responsible segment as the largest occupational area of tumor volume using thin CT (1–10 mm thickness). The primary endpoint was overall survival (OS) after pulmonary resection.

Because individual patients were not identified, our institutional review board (Review Board in Cancer Institute Hospital, Japanese Foundation for Cancer Research) approved this study without requiring patient consent. Patient records and information was anonymized and de-identified prior to analysis.

### Method to evaluate the therapeutic value of lymph node dissection

We used Sasako’s method to evaluate the therapeutic value of lymph node dissection according to the index of the benefit gained by dissection of each station [15]. This analysis was calculated by multiplying the frequency of metastasis to the station and the 5-year survival rate of patients with metastasis to the station [15].

### Statistical analysis

All data were analyzed using SPSS version 17.0 (SPSS Institute Incorporated, Chicago, Illinois, USA). Sensitivity and specificity were compared using standard formulae. Differences between two groups were calculated using the Mann–Whitney test, and comparisons between more than three groups were calculated using the Kruskal–Wallis test. Multivariate analyses were performed using the Cox proportional hazards model. Analysis of survival rates was performed using the Kaplan–Meier method, and comparison of survival between patient groups was performed via a log-rank test. *p <* 0.05 was considered to indicate statistical significance.

## Results

### Descriptive statistics

Patient characteristics are summarized in Table 1. In total, 417 eligible patients were enrolled; 237 men and 180 women, with a mean age of 63 years (26–84 years). The median follow-up duration was 1641 days (37–5197 days). More tumors were located in the upper division of left upper lobe than in the lingular lobe, and the primary histological diagnosis was adenocarcinoma.

**Table 1:** Characteristics of patients with left upper lobe non-small cell cancer.

Variables	Lobe (n = 417)	Upper division (n = 348)	Lingula (n = 69)
Age in years			
Mean (Range)	63 (26–84)	63 (26–84)	63 (38–80)
Sex			
Male/Female	237/180	203/145	34/35
Histology			
Adenocarcinoma	325	272	53
Squamous cell carcinoma	52	45	7
Others	40	31	9
Segment			
Upper division	348		
Apicoposterior/anterior	228/120		
Lingula	69		
pN status			
N0	263	220	43
N1	70	58	12
N2	84	70	14

### Metastasis pattern according to tumor location

Table 2 summarizes the frequency of node involvement per station in the primary upper division and lingular NSCLC. Nodes 11 (#11; *p* < 0.01) and 7 (#7; *p* < 0.01) were significantly correlated with the difference of node involvement, but none of the other node stations showed significant difference by univariate analysis. We performed multivariate analysis to identify clinicopathological factors that had an important impact on the metastasis of #11 among pN2 patients, but no significant difference was observed in any variables such as age (>70, *p* = 0.41), smoking habits (BI > 400, *p* = 0.26), tumor marker [Carcinoembryonic antigen (CEA)>5, *p* = 0.72], tumor size (>30 mm, *p* = 0.11), pathology [adenocarcinoma (AD) or non-AD, *p* = 0.72], and differentiation index (poorly or not, *p* = 0.91). Node involvement to the upper mediastinal zone, including the lower paratracheal and aortic nodes, was predominantly observed in patients with a tumor located in the upper division; however, significant differences were not observed.

**Table 2:** Frequency of lymph node metastases.

Variables	Upper division (%) (n = 348)	Lingula (%) (n = 69)	*p*
pN1			
Main bronchus (#10)	22.7	15.4	0.41
Interlobar (#11)	23.4	53.8	<0.01^a^
Lobar (#12)	47.6	42.3	0.62
pN2			
Lower paratracheal (#4L)	19.5	7.7	0.15
Aortic (#5/6)	44.5	26.9	0.10
Subcarinal (#7)	3.9	26.9	<0.01^a^

^a^ *p* < 0.05

# indicates “lymph node number” as defined by the Committee of the International Union against Cancer [12].

### Predictive benefit of lymph node involvement in primary upper division NSCLC

**Fig 1:**
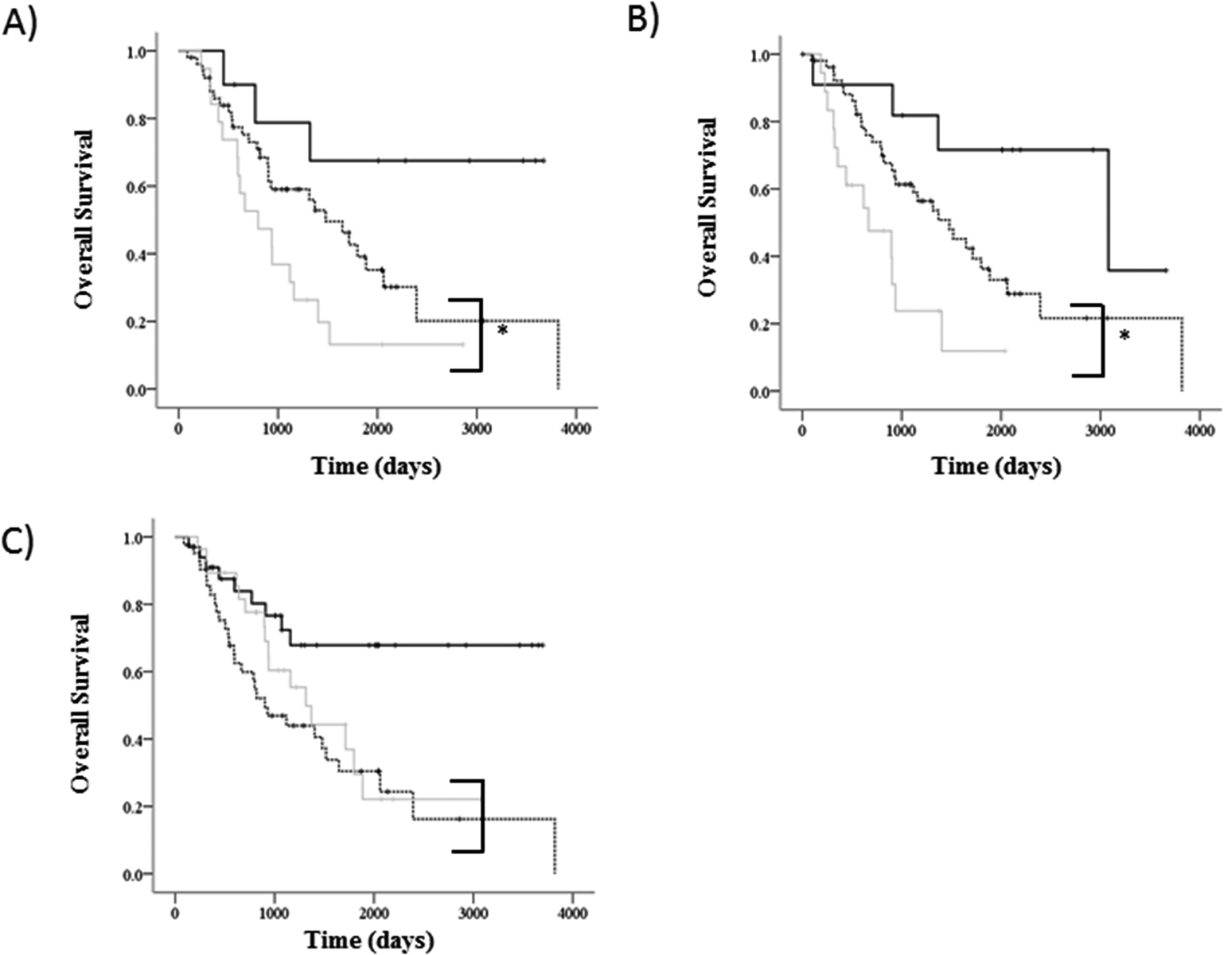
Kaplan–Meier graphs of overall survival. (a) Black line: #10(+)N2(−); dotted: #10(−)N2(+); gray #10(+)N2(+), (*p* = 0.01^*^). Parentheses indicates *p* = 0.04^*^. (b) Black line: #11(+)N2(−); dotted: #11(−)N2(+); gray #11(+)N2(+), (*p* < 0.01^*^). Parentheses indicates *p* = 0.01^*^. (c) Black line: #12U(+)N2(−); dotted: #12U(−)N2(+); gray #12U(+)N2(+), (*p*<0.01^*^). Parentheses indicates *p* = 0.38. ^*^ *p* < 0.05. # indicates “lymph node number” as defined by the Committee of the International Union against Cancer (12). (+) and (−) represent the positive and negative status of the node, respectively.

We evaluated the prognostic usefulness of node involvement in primary upper division NSCLC. The total pathological nodal statuses are summarized in Table 1, with most cases being pN0 (63.2%) and comparable numbers of pN1 and pN2 (16.7% and 20.1%, respectively). The 5-year OS in pN1 and the number of cases per group according to lymph node location were 31.5% for #10 (n = 29), 39.3% for #11 (n = 30), and 50.4% for #12U (n = 61) (Fig 1A, 1B, and 1C). The 5-year OS in pN2 and the number of cases per group according to lymph node location were 26.9% (n = 25) and 29.7% (n = 57) for lower paratracheal and aortic nodes, respectively. Multivariate analysis revealed that N2 lymph node metastases to both lower paratracheal (*p* = 0.01) and aortic nodes (*p* < 0.01) nodes were significant prognostic factors in survival (Table 3).

**Table 3:** Five-year overall survival for each nodal status in left upper division non-small cell lung cancer with multivariate analyses.

Variables	n (N1/N2)	OS (%)	*p*	HR	95% CI
pN1					
Main bronchus (#10)	29 (10/19)	31.5	0.06	1.71	0.98–2.97
Interlobar (#11)	30 (12/18)	39.3	0.19	1.49	0.81–2.74
Lobar (#12)	61 (33/28)	50.4	0.44	1.22	0.74–1.99
pN2					
Lower paratracheal (#4L)	25	26.9	<0.01^a^	2.11	1.17–3.79
Aortic (#5/6)	57	29.7	0.01^a^	3.41	2.02–5.76

CI; Confidence interval; HR: hazard ratio; OS: overall survival

^a^ *p* < 0.05

# indicates “lymph node number” as defined by the Committee of the International Union against Cancer [12].

### Therapeutic benefit of lymph node dissection in primary upper division NSCLC

**Fig 2:**
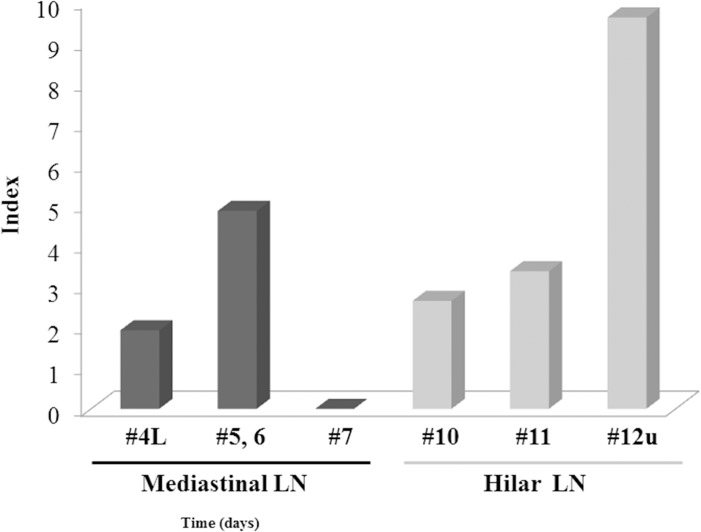
The therapeutic value of each lymph node dissection in left upper division non-small cell lung cancer.

The calculated values of each lymph node dissection in primary upper division are shown in Fig 2. The therapeutic index of estimated benefit from lymph node dissection for #11 was approximately equal to that for #10, both of which belong to the hilar/interlobar zone, according to AJCC. The therapeutic index for #11 was superior than that for the upper zone (0.57-fold) and the subcarinal zone (0-fold), but inferior than that for the AP zone (1.44-fold).

### Prognosis of hilar lymph node metastasis (N1) in primary upper division NSCLC

We examined the distribution and prognosis of patients with N1 involvement (n = 58). The N1/N2 populations were 34.5%, 40.0%, and 54.1% for #10, #11, and #12U, respectively. We divided each N1 (i.e., #10, #11, and #12U) location into three categories as follows: N1(+)N2(−), N1(−)N2(+), and N1(+)N2 (+). The 5-year OS rates for #10 involvement were 67.5% for #10(+)N2(−), 39.1% for #10(−)N2, and 13.2% for #10(+)N2; the number of cases per group were 10, 59, and 19, respectively (Fig 1A; *p* = 0.01). The 5-year OS rates for #11 involvement were 71.6% for #11(+)N2(−), 36.3% for #11(−)N2, and 11.9% for #11(+)N2; the number of cases per group were 12, 52, and 18, respectively (Fig 1B; *p* < 0.01). The 5-year OS rates for #12U involvement were 67.8% for #12U(+)N2(−), 30.4% for #12U(−)N2, and 29.5% for #12U(+)N2; the number of cases per group were 33, 42, and 28, respectively (Fig 1C; *p* < 0.01).

Multivariate analysis revealed that #11 involvement was an independent prognostic indicator in the survival of patients with pN2 [*p* = 0.02, hazard ratio (HR) = 2.29, 95% confidence interval (CI) = 1.15–4.55] (Table 4). The frequency of lymph node metastasis to each region in primary upper division NSCLC with #11 involvement was as follows: for N1, #10 was 38.9% (n = 7) and #12U was 61.1% (n = 11), whereas for N2, #4L was 55.6% (n = 10), #5/6 was 88.9% (n = 16), and #7 was 16.7% (n = 3). A significant difference was found between the upper mediastinal zone and lower subcarinal zone (*p* < 0.01) (Table 5). The frequency of #11 involvement was 1.89-fold higher in the anterior segment (12.5%, 15/120) than that in the apicoposterior segment (6.6%, 15/228), but the difference was not significant (*p* = 0.06).

**Table 4:** Multivariate analysis for pN2 patients in left upper division non-small cell lung cancer according to each N1 status.

Name	n	*p*	HR	95% CI
pN1				
Main bronchus (#10)	19	0.17	1.59	0.82–3.03
Interlobar (#11)	18	0.02^a^	2.29	1.15–4.55
Lobar (#12U)	28	0.40	0.77	0.40–1.46

CI: Confidence interval; HR: hazard ratio

^a^ *p* < 0.05

# indicates “lymph node number” as defined by the Committee of the International Union against Cancer [12].

**Table 5:** Distribution of node involvement in pN2 patients in upper division non-small cell lung cancer with interlobar node metastases.

Lymph nodes	Number (n = 18)	%
pN1		
Main bronchus (#10)	7	38.9
Lobar (#12)	11	61.1
pN2		
Lower paratracheal (#4L)	10	55.6
Aortic (#5/6)	16	88.9
Subcarinal (#7)	3	16.7

Finally, we also performed multivariate analysis to identify whether the metastasis of #11 could be an independent negative prognostic factor among pN2 in some variables. Tumor marker (CEA>5, *p* = 0.03), and nodes involvement of {#11 (p = 0.01), and #12 (*p* < 0.01)} were revealed to be independent negative prognostic factors affecting OS, and other variables such as sex (*p* = 0.78), age (>70, *p* = 0.84), smoking habits (BI > 400, *p* = 0.34), tumor size (>30 mm, *p* = 0.30), pathology [adenocarcinoma (AD) or non-AD, *p* = 0.27], differentiation index (poorly or not, *p* = 0.54), and node involvent of #10 (p = 0.55) were not.

## Comment

The standard treatment procedure for NSCLC involves lobectomy with radical lymphadenectomy [16, 17]. There has been a trend to use various surgical procedures, including extremely limited procedures, such as wedge resection, anatomical segmentectomy, and selective mediastinal lymph node dissection, in highly selected early NSCLCs [10]. In addition, Nomori et al. reported in their single institution prospective cohort study that limited surgery with radical lymph node dissection contributed to improved survival when using sentinel lymph node detection for selected cT1N0M0/pN0 NSCLC [11]. Moreover, another study indicated that nodes #10, #11, #12, and #13 were sentinel nodes in 10%, 16%, 39%, and 57% of cases, respectively [18]. To prevent the overgeneralization of this strategy, we reviewed lymph node spread and interlobar lymph node status associated with left upper lobe NSCLC patients only. We previously reported that preoperative factors associated with lymph node metastases mediastinal window setting on CT were 0% for <10 mm (n = 52), and the incidence of ly/v/pl was 0% ≤5 mm and 25.9% (n = 27) for >5 mm to 10 mm (n = 52) [19]. Therefore, we proposed that mediastinal diameter may be a promising criteria for CT classification as the newly proposed minimally invasive adenocarcinoma [19]. Thus, the applicable population that could be matched to our new proposal criteria with limited resection is naturally selected. Our aim was to evaluate whether it is appropriate to omit interlobar lymph node dissection in left upper division segmentectomy on the basis of the data obtained from two cancer-specialized centers.

Anthony et al. reviewed that left upper lobe tumors have a high incidence of non-regional metastasis, and therefore, the drainage pattern of lingula is different [20]. Asamura et al. reported that 34 left upper segment tumors most commonly metastasized to the aorticopulmonary window (APW; 71%), followed by the para-aortic station (32%) and the subcarina (12%), whereas the 10 left lingular tumors metastasized to the subcarina, most commonly (50%) followed by APW (20%) [20]. Our cohort showed similar patterns of metastatic lymphatic spread between the upper division and lingula; however, metastases to the upper mediastinal zone (i.e., lower paratracheal and aortic nodes) were no more frequent in the upper division than that in lingula. We further studied N1 lymph node metastases by this difference of lymphatic flow and determined that upper division NSCLC metastasis to interlobar nodes was significantly lower than that from lingula (*p* < 0.01); however, the incidence of interlobar nodal metastasis was >20%. Therefore, we concluded that metastasis to interlobar lymph nodes may not play an important role considering that it exceeded 20%.

In our study, left upper division NSCLC frequently metastasized to interlobar nodes (23.4%) despite the pathological type (adenocarcinoma or not, *p* = 0.89). The population of pN1 was higher in patients with upper division NSCLC and interlobar node involvement (40%, 12/30). If interlobar lymph node dissection had not been performed, 9.4% patients with upper division NSCLC would have been pathologically diagnosed as having no nodal involvement. The concept of adjuvant chemotherapy is established by many clinical trials and contributes to prolonged survival in patients with lymph node involvement [1–3]. The omission of interlobar lymph node dissection during upper division segmentectomy may be associated with incorrect pathological staging, and therefore, an opportunity to benefit from adjuvant chemotherapy is lost. By multivariate analysis, interlobar lymph node metastasis was inferior to mediastinal lymph node metastasis as a prognostic factor. We conclude that interlobar node dissection may improve staging and ensure appropriate treatment for patients with upper division NSCLC. We also examined whether it was intentionally regarding each lymph node dissection using Sasako’s method [15]. The high therapeutic index for #11 following AP zone that form lymph node dissection according to Sasako’s scale may support our claim in left upper division NSCLC.

Our study was consistent with other reports in that the prognosis of NSCLC patients with mediastinal lymph node metastases was significantly worse than those with only N1 nodes [21]. However, we also examined whether the path of lymph node spread impacted the prognosis. In patients with mediastinal lymph node metastases, only lobar lymph nodes failed to show significant differences (*p* = 0.38); in other words, no lymph node involvement around the main bronchus (i.e., the hilar and interlobar lymph nodes) was statistically associated with a better prognosis in patients with mediastinal lymph node metastasis (*p* = 0.04 and *p* = 0.01, respectively). Okada et al. reported that lymph nodes around the main bronchus could be designated as intermediate and there may be no borderline between N1 and N2 nodes around the main bronchus, based on the clinical records of equivalent prognoses between hilar N1 disease and N2 single-station disease [22]. Our result was compatible with this finding despite the difference in the lobe. We also postulated that lymphatic flow via the interlobar node to the mediastinum has a worse prognosis by univariate analysis and the independent prognostic factor by multivariate analysis in the NSCLC patients with mediastinal lymph node metastases (Table 4, Fig 1). We concluded that interlobar node dissection was an essential intraoperative component of both left lingular division and left upper division NSCLS.

To attempt left upper division segmentectomy, incomplete dissection for interlobar lymph node may occur because of variations in the divergence style of the lingular artery and vein. Interlobar node dissection in left upper division segmentectomy is technically more challenging and requires longer operative time, particularly when performed via a thoracoscopic approach. The interlobar nodes are positioned behind lingular segmental veins from an anterior mediastinal view, and behind the lingular segmental arteries from an interlobar fissure view. The formation of an interlobar fissure, while trying to avoid impairment of these vessels, is a necessity of interlobar node dissection. We modified our operative strategy for left upper segmentectomy to reflect this retrospective study since November 2012. In the surgical experience at our institutions, in comparison with conventional surgery, additional interlobar node dissection has not been associated with an increase in postoperative complications. We aim to perform a further prospective study to confirm our results in the near future.

There are some limitations to this study; although the main issue is arguably its retrospective design, the number of participants included was acceptable. Furthermore, to avoid institutional bias, this study was undertaken at two cancer-specialized centers. However, a multi-institutional cohort that included different strategies would be superior; in our study, thoracic surgeons at the two participating institutions utilized the same strategy for radical lymphadenectomy. In addition, this study was based on lobectomy with thorough lymphadenectomy employed for standard pulmonary resection. To provide meaningful data on prognostic differences, a future prospective study is required, and is in the planning phase.

In conclusion, our study suggests that interlobar node involvement from left upper division NSCLC is not rare, occurring in over 20% cases. However, significant differences exist between upper division and lingula NSCLC. In addition, we demonstrated that metastasis via the interlobar node is an independent poor prognostic factor in patients with N2 disease. It is reasonable to conclude that dissection of the interlobar lymph node is as important as that of the mediastinal lymph node during left upper radical segmentectomy, and may contribute to accurate pathological staging and appropriate treatment.
